# A systematic review and meta-analysis of the association between cyproterone acetate and intracranial meningiomas

**DOI:** 10.1038/s41598-022-05773-z

**Published:** 2022-02-04

**Authors:** Keng Siang Lee, John J. Y. Zhang, Ramez Kirollos, Thomas Santarius, Vincent Diong Weng Nga, Tseng Tsai Yeo

**Affiliations:** 1 Bristol Medical School, University of Bristol, Bristol, UK; 2 Yong Loo Lin School of Medicine, National University of Singapore, Singapore, Singapore; 3 Division of Neurosurgery, Department of Clinical Neurosciences, University of Cambridge and Addenbrooke’s Hospital, Cambridge, UK; 4 Department of Neurosurgery, National Neuroscience Institute, Singapore, Singapore; 5 Division of Neurosurgery, Department of Surgery, National University Hospital, National University Health System, Singapore, Singapore

**Keywords:** Drug safety, Surgical oncology, Risk factors

## Abstract

The influence of exposure to hormonal treatments, particularly cyproterone acetate (CPA), has been posited to contribute to the growth of meningiomas. Given the widespread use of CPA, this systematic review and meta-analysis attempted to assess real-world evidence of the association between CPA and the occurrence of intracranial meningiomas. Systematic searches of Ovid MEDLINE, Embase and Cochrane Controlled Register of Controlled Trials, were performed from database inception to 18th December 2021. Four retrospective observational studies reporting 8,132,348 patients were included in the meta-analysis. There was a total of 165,988 subjects with usage of CPA. The age of patients at meningioma diagnosis was generally above 45 years in all studies. The dosage of CPA taken by the exposed group (n = 165,988) was specified in three of the four included studies. All studies that analyzed high versus low dose CPA found a significant association between high dose CPA usage and increased risk of meningioma. When high and low dose patients were grouped together, there was no statistically significant increase in risk of meningioma associated with use of CPA (RR = 3.78 [95% CI 0.31–46.39], p = 0.190). Usage of CPA is associated with increased risk of meningioma at high doses but not when low doses are also included. Routine screening and meningioma surveillance by brain MRI offered to patients prescribed with CPA is likely a reasonable clinical consideration if given at high doses for long periods of time. Our findings highlight the need for further research on this topic.

## Introduction

Meningiomas are typically slow growing benign tumors arising from the meningothelial cells of the arachnoid membrane encasing the central nervous system^1,2^. Ninety percent of meningiomas are intracranial, and they account for 38% of all intracranial tumors reported in the United States (US) between 2013 and 2017^3^. These tumors are often revealed incidentally by imaging. When symptoms arise, it is the result of raised intracranial pressure, which vary according to the size and location of the tumor.

The etiology of meningiomas is controversial but unequivocal risk factors are environmental or medical exposure to ionizing radiation^4–6^, and hereditary mutations of the neurofibromatosis type 2 gene^7–10^. Strong evidence also suggests a plausible role for sex hormones in meningioma development. These include the predilection for females especially after puberty^3^, and the well characterized distribution of progesterone, estrogen, and androgen receptors in certain skull base meningiomas^11–18^. Furthermore, fluctuations in meningioma growth during the menstrual cycle, pregnancy, and breastfeeding have also been well-documented^19–26^. Benson et al., in a meta-analysis demonstrated that the use of hormone replacement therapy is an independent risk factor for the development of meningiomas^26^.

Given the hormone-sensitive nature of meningiomas, the influence of exposure to hormonal treatments, particularly cyproterone acetate (CPA), has been theorized to contribute to the growth of meningiomas. CPA is a synthetic progestogen with potent anti-androgenic, progestogenic and antigonadotrophic mechanistic actions^27,28^. The dose and indications for CPA vary considerably. High dose CPA formulations (> 50 mg/day) are used in persons of male birth sex with inoperable prostate cancer, paraphilia, hirsutism, or male-to-female transsexual hormonal therapy^27^. Lower doses (2-10 mg/day) are used in combination with estradiol for birth control as well as to treat androgen-associated alopecia or female seborrhea^28^.

The first signal of an association of prolonged use of high dose CPA with meningioma was raised in a transsexual patient reported by Gazzeri et al.^29^. In this case, a causal association between the abrupt growth of a giant grade 1 olfactory-groove meningioma and the hormone therapy was suggested by the negative cerebral MRI scan obtained three years before presentation. Since then, several case series^30–36^, and adequately powered cohort studies have corroborated these findings^37–40^. The presence of progesterone receptors on meningiomas supports the biological plausibility of an association. Furthermore, previous robust in vitro and preclinical studies support the efficacy of progesterone receptor antagonist such as mifepristone (RU 486) in meningiomas^41–44^, which supports the argument regarding a causal relationship.

Given the widespread use of CPA, any plausible drug-related risk of meningiomas should be investigated thoroughly. The main objective of this present study was to appraise real-world evidence of the association between CPA and the occurrence of intracranial meningiomas.

## Results

### Study selection and characteristics

**Figure 1:**
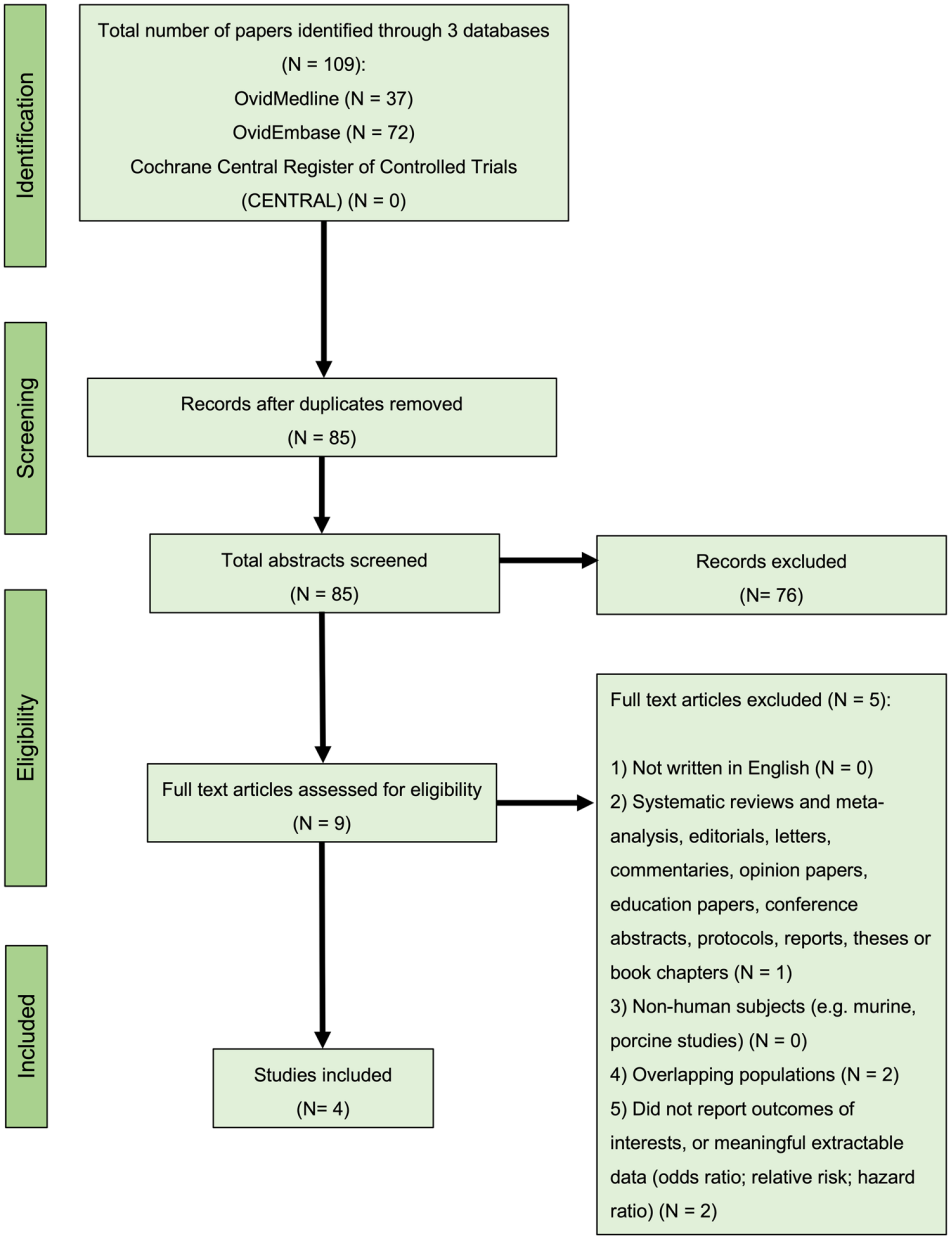
PRISMA flow diagram for studies included and excluded from the systematic review and meta-analysis.

Figure 1 presents the Preferred Reporting Items for Systematic Reviews and Meta-Analyses (PRISMA) flow diagram illustrating the number of reviews screened and reasons for exclusion at each stage. Using the designated search terms, a total of 109 articles were retrieved, and four were included in the final dataset^37,40,45,46^. There were three publications analyzing the same cohort, and the data by Weill et al.^37^ was chosen over the other two^38,39^, as it reported the largest patient-year data. Similarly, two publications had analyzed overlapping data from Danish healthcare registers and the more recent study by Mikkelsen et al.^46^, with larger patient-year data was chosen over the other^47^. Reliability of study selection between observers was substantial at both the title and abstract screening stage (Cohen’s κ = 1.00) and the full-text review stage (Cohen’s κ = 1.00)^48^.

All four included studies were retrospective^37,40,45,46^. Three were cohort studies and one was a case–control study^40^. The three cohort studies both adopted nation-wide population-based databases (from Denmark, France and Spain)^37,45,46^. The case–control study identified cases and controls from a large UK primary care database^40^. Controls were selected at random and frequency-matched to cases by age (within one year), sex and index year (year of newly diagnosed meningioma). Table 1 summarizes the baseline characteristics and outcomes in each included study.

**Table 1:** Summary of the baseline characteristics and outcomes in each included study.

Study	Country	Database	Study period	Type of study	Patient sample size	Females	No. of exposed	No. of current use	No. of past use	No. of high dose	No. of low dose	No. of non-exposed	Definition of exposed	Definition of high dose exposure	Definition of low dose exposure	Exposed with meningioma	Exposed with meningioma requiring neurosurgery/radiotherapy	High dose exposure and meningioma	Low dose exposure and meningioma	Non-exposed with meningioma	Location of CPA-associated meningioma breakdown
Cea-Soriano^40^	UK	The Health Improvement Network (THIN) UK primary care database	Jan 1996 to June 2008	Retrospective case–control study	10,745	7896	72	26	46	16	56	10,673	For females: all had ≥ 2 mg/day CPA in combination with estrogens; For males: all had ≥ 50 mg/day + recorded diagnosis of prostate cancer Exposure to drugs was classified as: (1) ‘current use’, where the most recent prescription lasted until the index date or ended in the year before the index date; (2) ‘past use’, when the most recent use was more than 1 year before the index date; and (3) ‘non-use’, when there was no recorded use of the drug at any point before the index date	Daily dose 50 mg or higher	All daily doses < 50 mg	8	NA	4	4	737	NA
Gil^45^	Spain	Base de datos para la Investigación Farmacoepidemiológica en Atención Primaria (BIFAP) database	Jan 1, 2001 to Dec 31, 2007	Retrospective cohort study	2,137,191	NA	24,712	NA	NA	2474	22,238	2,112,479	All patients receiving at least one high dose (50 mg) CPA prescription during their follow-up	Daily dose 50 mg or higher	All daily doses < 50 mg	4	NA	4	0	452	NA
Mikkelsen^46^	Denmark	Danish prescription register; National patient register, Cancer register	1997 to 2019	Retrospective cohort study	5,730,635	NA	1982	NA	NA	781	1201	5,728,653	Cumulative dose of CPA was summed during the follow-up and recipients were categorised into three exposure groups: no CPA, 0.1–10 g of CPA (obtained after the first prescription of CPA), > 10 g of CPA	> 10 g at end of follow-up	< 0.1–10 g at end of follow-up	16	NA	10	6	8940	NA
Weill^37^	France	French administrative health care database (SNDS)	2007 to 2014	Retrospective cohort study	253,777	253,777	139,222	NA	NA	NA	NA	1,145,555	Cumulative dose was greater than or equal to 3 g (at least three standard packs of 20, 50 mg tablets) within the first 6 months of this first prescription	NA	NA	69	NA	NA	NA	20	Anterior skull base, n = 190 (36.8%) Middle skull base, n = 130 (25.2%) Posterior skull base, n = 20 (3.9%) Convexity, not involving dural venous sinuses, n = 107 (20.7%) Convexity, involving dural venous sinuses, n = 19 (3.7%) Falx and tentorium, n = 29 (5.6%)

*NA* not applicable.

### Quality assessment

Using the Joanna Briggs Institute (JBI) checklist for prevalence studies, three studies attained a full score of 11 and one attained a score of 10 (Supplementary Table 3).

### Patient characteristics

A total of 8,132,348 patients were reported across the four included studies^37,40,45,46^. Patient gender was reported in two studies, of which 261,673 of the total 264,522 patients were females (98.9%)^37,40^. There was a total of 164,006 subjects with usage of CPA. The age of patients at meningioma diagnosis was generally above 45 years in all studies. In the study by Cea-Soriano et al., the mean age at meningioma diagnosis was 62.6 and 62.2 years for female and male patients, respectively^40^. Gil et al. reported that 403 out of 456 (88.4%) meningioma patients were above the age of 45 years^45^. Similarly, Weill et al. reported a mean age of 48.1 and 50.5 years at meningioma diagnosis for the exposed and control groups, respectively^37^.

### Exposure and dosages

The dosage of CPA taken by the exposed group (n = 165,988) was specified in three of the four included studies. In the study by Cea-Soriano et al., all female patients had a daily CPA dose of 2 mg or higher, whereas all male patients had a daily dose of 50 mg or higher^40^. In the study by Weill et al., the cumulative dose of patients within the exposed group was greater than or equal to 3 g (at least three standard packets of 20, 50 mg tablets) within the first six months of the first prescription^37^. The studies by Cea-Soriano et al. and Gil et al. defined high dose as ever having a daily dose of 50 mg or higher, while low dose was defined in these studies as all daily doses being less than 50 mg, at a markedly lower dose of 2 mg/day (which may likely be for birth control)^40,45^. The study by Mikkelsen et al., compared the incidence of intracranial meningiomas between groups of high cumulative doses (> 10 g) versus low cumulative doses of CPA (0.1–10 g). Across the three studies (with total sample size being 7,851,805 and number of exposed patients being 26,766), there were a total of 3271 and 23,495 high and low dose patients, respectively^40,45,46^.

### Risk of meningioma associated with use of CPA

All four studies report an increased risk of meningioma associated with high doses of CPA exposure^7,40,45,46^.

Cea-Soriano et al., Gil et al. and Mikkelsen et al., demonstrated an increased risk of meningioma with use of high dose CPA (defined as above) compared to non-users and use of low dose CPA^40,45,46^. The distinction between current and past users of CPA was reported in the study by Cea-Soriano et al. and Mikkelsen et al., but not specified in the one by Gil et al. Cea-Soriano et al. found that there was no significantly increased risk of meningioma with past use of CPA, as well as current or ever use (which includes both current and past use) of low dose CPA^40^. Mikkelsen et al., on the other hand, showed significantly increased risk of meningioma with past and present use of CPA, compared with no use^46^.

Similarly, Weill et al. found a dose–effect relation between meningioma risk and cumulative dose of CPA, with higher risk associated with a higher cumulative dose^37^. The hazard ratio (HR) was not significantly different from 1 for exposure to less than 12 g of CPA, and it rapidly increased for higher cumulative doses: 11.3 (95% CI 5.8–22.2) for 36–60 g and 21.7 (95% CI 10.8–43.5) for 60 g or higher. In this study, the exposed group comprised only of current users and does not include past users^37^.

**Figure 2:**
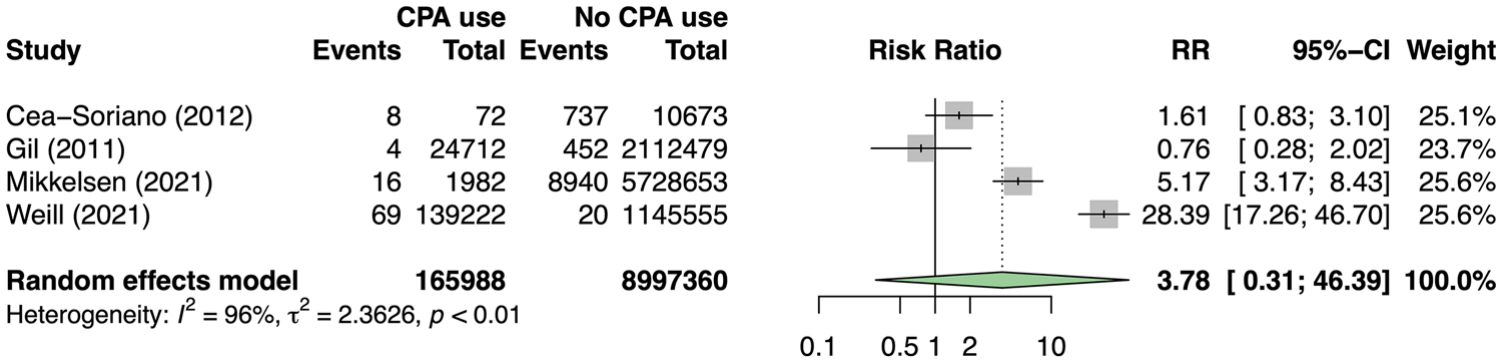
Forest plot demonstrating the association between CPA use and intracranial meningioma.

We pooled the patients across the four included studies to perform a meta-analysis of binary outcome. The total number of patients in the exposed and non-exposed group was 165,988 and 8,997,360, respectively. Meta-analysis demonstrated no statistically significant increased risk of meningioma associated with use of CPA (risk ratio [RR] = 3.78 [95% CI 0.31–46.39], p = 0.190) [Fig. 2]. Study heterogeneity was substantial and statistically significant (I^2^ = 95.7% [95% CI 91.9–97.8], p < 0.001).

### Anatomical location of meningioma

Only Weill et al. reported the anatomical location of CPA-associated meningiomas and hence a pooled subgroup analysis was not possible for anatomical location. Weill et al. demonstrated that the risk of CPA-associated meningioma varied considerably according to their anatomical locations, with a predilection for the anterior base of the skull base (RR 43.6 [95% CI 13.9–137.1] and adjusted HR 47.1 [95% CI 14.9–149.1]).

## Discussion

To our knowledge, this is the first meta-analysis to investigate the association between CPA use and intracranial meningioma. Limited current evidence suggests an increased risk of meningioma associated with high dose CPA usage. When high dose users were combined with low dose users, this association becomes statistically insignificant. This meta-analysis underscores the current paucity in evidence about the risk of intracranial meningioma associated with low dose CPA. For example, for the purposes of birth control prescribed at 2 mg or 50 mg for short periods. The included studies had also varied in their definition of low dose CPA prescription. It is still unknown whether or not CPA below a certain threshold may be safe in terms of the risk of meningioma.

### Location

A majority of CPA-associated meningiomas have been reported to be preferentially distributed at the anterior (22–75%) and middle base of the skull (25–40%), as opposed to cranial convexity which is the commonest location in the general population^16,32,37–39^. Samarut and colleagues purported that meningiomas located in the anterior and middle skull base appeared to be specific to CPA use, with the risk reducing after termination of CPA^38^. The predominance of anterior skull base meningiomas may be supported by biological plausibility^49^. The embryological biology of meninges differs at the convexity (neural crests) and the base of the skull (mesoderm)^16,32,50–56^. Molecular and immunohistochemical studies have established that the progesterone receptor distribution in the skull base follows a rostrocaudal gradient^14–16,57^. Thus, we could expect skull base meningiomas to dominate in the anterior cranial fossa with progestogenic CPA exposure.

### Risk, causality and interpretation

A causal relationship between high dose CPA and the development of meningioma is tenable. Based on the Bradford Hill criteria^58^, this may be supported by the strength and dose-dependent association. Our findings suggest a modest magnitude of the association between high dose CPA use and intracranial meningiomas, albeit when high dose users were banded together with low dose users in our pooled analysis, this association became statistically insignificant. Although a three-fold increase in clinically significant risk was found in our meta-analysis, the confidence intervals encompassed the null. This is further supported by the specificity of certain tumor locations (anterior skull base) which are highly dense with progesterone receptors, providing a biological plausibility.

Reverse causality is acknowledged with observational studies especially if the prescription of CPA was linked to an undiagnosed meningioma. However, this bias may be excluded from our meta-analysis because of the temporal aspect of our findings: the risk of meningioma increased with the duration of CPA use and cumulative doses, and not during the initial phase of drug use. Furthermore, reports of rapid spontaneous meningioma regression or stabilization after CPA withdrawal, can be found in the literature^30,31,33,36,59^. This observation further reinforces the notion of causality^58^. As progesterone have been postulated to accelerate meningioma growth by vascularization, the biology involved is analogous to the spontaneous regression of meningiomas postpartum^60^.

### Clinical implications and management of CPA-associated meningiomas

Iatrogenic meningioma engendered by high-dose CPA use is a public health issue. Before these results are used to guide clinical decision making, the collective body of data on this safety issue should be scrutinized by drug regulatory authorities and weighed against the benefits of treatment. Nonetheless, patients currently on or previously exposed to high dose CPA should be informed about the increased risk of intracranial meningiomas. The indication of CPA should be clearly defined with the lowest possible daily dose used.

First line management of meningiomas typically involves surgery. Location of the meningioma influences the extent of resection, which, consequently influences outcomes such as recurrence rates^61^. As shown, CPA-associated meningiomas have a predilection for the skull base, which is of considerable importance because skull base meningioma surgery is associated with poorer prognosis than surgery for non-skull base meningiomas^61–64^. Duly, evidence for spontaneous meningioma regression with CPA termination^30,31,33,36,59^, sustained the notion that invasive treatment may be avoided and conservative management of CPA-associated meningiomas might be treatment of choice^30,39,65^. However, it must be noted that such cases are exceptional—a patient with clinoidal meningioma and progressive visual loss must be operated on, in spite of previous treatment with CPA. Conservative management, which may be recommended for small and asymptomatic meningiomas, comprises cessation of CPA and close follow-up magnetic resonance imaging (MRI) in the context of current or past history of high dose exposure. As this screening suggestion was not directly investigated in this study, this requires further cost–benefit analysis by guideline groups and/or policymakers. Despite evidence that antiprogesterone treatment reduces the size of meningioma, both in vitro and in vivo, such therapy has not been recommended in the conservative management of meningiomas.

### Limitations

Although several factors lend support to the strength of the association, including biological plausibility and consistent epidemiological evidence, our findings must be cautiously interpreted in the context of its known limitations. Limitations of our meta-analysis include the retrospective and observational nature of included studies and the significant heterogeneity among the studies. There were no randomized controlled trials in this study, although conducting one could account for potential biases and confounders, the non-randomized evidence to the risk of meningiomas is so extensive that this would unlikely take place, from practical and ethical standpoints^66^. A further limitation of the available data is that there is little known about the impact of past exposure or whether there is a cumulative dose effect, and hence we were unable to weigh the effect of historical doses versus current doses differently. Only two studies had defined past exposure^40,46^. Confounding factors are inevitable in any of our included observational studies. The small number of studies available in the literature could explain the finding of non-significance and limited our ability to perform certain analyses such as meta-regression to explore possible confounders (age and sex) or sources of heterogeneity in our dataset. To minimize the extent of these limitations, we performed sensitivity analyses to attempt to identify outlier studies. Taken together in this light, together with our pooled analysis, we propound that this relationship cannot be proven causal given the aforementioned. Nonetheless, advantages of our meta-analysis include avoiding undue emphasis on individual studies, thus yielding risk estimates that are more reliable.

## Conclusion

In light of these results, prescription of high-dose CPA, especially for off label indications, should be considered carefully. Additionally, routine screening and meningioma surveillance by brain MRI offered to patients prescribed with CPA is likely a reasonable clinical consideration if given at high doses for long periods of time. The results obtained herein suggest the necessity for further clinical research on intracranial meningioma associated with CPA.

## Methods

The review was conducted according to the PRISMA guidelines^67^. The protocol was registered on the PROSPERO international prospective register of systematic reviews (registration number CRD42021242120).

### Search strategy

Searches of the following three electronic databases were undertaken: Ovid Medline, Ovid Embase, and Cochrane Central Register of Controlled Trials (CENTRAL).

Searches were performed in each database from its inception until 18th December 2021. The concepts of “cyproterone acetate”, and “meningioma”, were used in addition to synonyms and related terms. An example search strategy used for OVID Medline/EMBASE/CENTRAL is presented in Supplementary Table 1.

### Eligibility criteria

Any randomized or non-randomized study (cohort study; case–control study) that investigated the association between CPA use regardless of indication, and the risk of intracranial meningiomas were included. As it is the progestogenic effect of CPA that has been purported to contribute to intracranial meningiomas, the controls in this study were limited to patients unexposed to CPA or patients only very slightly exposed who discontinued CPA prematurely, as defined by the included studies. Particularly, in the study by Weill et al. the control group was defined as patients who discontinued treatment rapidly after having received a cumulative dose less than 3 g (one or two standard packs) dispensed within the first six months after this first prescription.

The following designs were excluded: case reports/series; non-English; animal studies. Studies that did not report extractable data including odds ratio (OR), RR, HR, or raw data, were also excluded. Patients were included regardless of gender and ethnicity, or presence of symptoms on presentation. Supplementary Table 2 describes the full list of inclusion and exclusion criteria.

### Study selection

All titles and abstracts were screened against the pre-defined eligibility criteria developed independently by two reviewers (KSL and JJYZ). Disagreements were resolved by discussion, and where agreement could not be reached, the senior reviewer assisted with decision making (VDWN). Potentially eligible studies were selected for full-text analysis. At each stage, KSL and JJYZ reviewed 100% of the screened studies for inclusion to ensure reliability of study selection. Disagreements were resolved by consensus or appeal to a third senior reviewer (VDWN). Agreement among the reviewers on study inclusion were evaluated using Cohen’s kappa^48^.

In the event of multiple publications analyzing the same cohort, the publication that reported the largest patient-year data will be used for evaluation.

The reference lists of included studies were also scrutinized to pursue references of identified citations, in an effort to identify high quality resources in obscure locations that could have been overlooked in our search strategy^68^.

### Risk of bias assessment

The quality of included studies was assessed using the JBI checklist for cohort studies^69^. In summary, these tools rated the quality of selection, measurement and comparability for all studies and gave a score for cohort studies (maximum of 11). Two researchers (KSL and JJYZ) assessed the quality of all included studies and discussed discrepancies until consensus is reached.

### Outcome

The primary outcome of interest was the development of intracranial meningiomas amongst patients who have taken CPA.

### Data extraction

A pro forma was developed and piloted to extract data on the following variables to ensure standardization and consistency in this process: (1) study details, (2) study design, (3) participant demographics, (4) country and dataset, (5) selection criteria, (6) controls, (7) indication for CPA, (8) dose of CPA, (9) unadjusted HR or RR or OR, propensity-score adjusted HR, propensity-score matched HR, and covariate-adjusted HR. Two reviewers (KSL and JJYZ) independently and blindly extracted 100% of the data each to ensure reliability. Discrepancies or disagreements about extracted material were resolved by the senior reviewer (VDWN).

Where data was incomplete (e.g. outcomes of interest reported but not specific to CPA exposure), the study authors were contacted via email to obtain full data and were given two weeks to respond.

### Statistical analysis

A meta-analysis of binary outcomes was performed to compare the risk of meningioma between the exposed and non-exposed groups. The overall summary estimate was presented as a RR with its 95% confidence interval (CI), and was computed following a weighted analysis of the RR from each individual study. The random effects model was used to account for study heterogeneity, with the overall pooled estimate computed using the inverse variance method. CI for individual studies were calculated using the Wilson Score confidence interval method with continuity correction. The I^2^ statistic was used to present between-study heterogeneity, where I^2^ ≤ 30%, between 30 and 50%, between 50 and 75%, and ≥ 75% were considered to indicate low, moderate, substantial, and considerable heterogeneity, respectively^70^. P values for the I^2^ statistic were derived from the chi-squared distribution of Cochran Q test.

All statistical analyses were performed using R software version 3.4.3 (R Foundation for Statistical Computing, 2016). P-values less than 0.05 were considered statistically significant.

### Ethical approval

Ethical approval was not required for this systematic review and meta-analysis.

## Supplementary Information

Supplementary Information.

## Abbreviations

CI: Confidence interval
CPA: Cyproterone acetate
HR: Hazard ratio
OR: Odds ratio
PRISMA: Preferred reporting items for systematic reviews and meta-analyses
RR: Risk ratio
US: United States
