# Supplementary material for: A systematic review and meta-analysis of the association between cyproterone acetate and intracranial meningiomas
Source: Sci Rep. 2022 Feb 4;12:1942. doi: 10.1038/s41598-022-05773-z (PMC8816922; doi:10.1038/s41598-022-05773-z)
Supplement: Supplementary file 1 — Supplementary Information. [file 41598_2022_5773_MOESM1_ESM.docx]

Supplementary Table 1. Search strategy used for the three electronic databases on 18^th^ December 2021

| **OVID Medline search** | | **37 articles** | |
| --- | --- | --- | --- |
| No. | Search term | | |
| **Intracranial meningioma concept** | | | |
| 1 | exp Meningioma/ | | |
| 2 | Meningioma*.tw. | | |
| 3 | 1 or 2 | | |
| **Cyproterone acetate concept** | | | |
| 4 | exp Cyproterone acetate/ | | |
| 5 | Cyproterone acetate*.tw | | |
| 6 | 4 or 5 | | |
| **Combined concepts** | | | |
| 7 | 3 and 6 | | |
| **EMBASE search** | | | **72 articles** |
| No. | Search term | | |
| **Intracranial meningioma concept** | | | |
| 1 | exp Meningioma/ | | |
| 2 | Meningioma*.tw. | | |
| 3 | 1 or 2 | | |
| **Cyproterone acetate concept** | | | |
| 4 | exp Cyproterone acetate/ | | |
| 5 | Cyproterone acetate*.tw | | |
| 6 | 4 or 5 | | |
| **Combined concepts** | | | |
| 7 | 3 and 6 | | |
| **Cochrane Central Register of Controlled Trials (CENTRAL**) | | **0 articles** | |
| No. | Search term | | |
| **Intracranial meningioma concept** | | | |
| 1 | MeSH descriptor: [Meningioma] explode all trees | | |
| 2 | (Meningioma*.):ti,ab,kw | | |
| 3 | #1 or #2 | | |
| **Cyproterone acetate concept** | | | |
| 4 | MeSH descriptor: [Cyproterone Acetate] explode all trees | | |
| 5 | (Cyproterone acetate*):ti,ab,kw | | |
| 6 | #4 or #5 | | |
| **Combined concepts** | | | |
| 7 | #3 and #6 | | |

Supplementary Table 2. Inclusion and exclusion criteria used to select studies for the review

| Inclusion criteria | Exclusion criteria |
| --- | --- |
| Primary interventional or observational studies investigating the association between cyproterone acetate use, and the risk of intracranial meningiomas | - Not written in English - Systematic reviews and meta-analysis, editorials, commentaries, opinion papers, letters*, education papers, conference abstracts, protocols, reports, theses or book chapters - Non-human subjects (e.g. murine, porcine studies) - Overlapping populations - Did not report outcomes of interests, or meaningful extractable data (odds ratio; relative risk; hazard ratio) |

*Letters to Editors with original research data reported were included.

Supplementary Table 3. Joanna Briggs Institute quality assessment checklist for prevalence studies.

|  |  |  | | **Question no.** | | | | | | | | | | |  |
| --- | --- | --- | --- | --- | --- | --- | --- | --- | --- | --- | --- | --- | --- | --- | --- |
| **Study** | **1** | | **2** | | **3** | **4** | **5** | **6** | **7** | **8** | **9** | **10** | **11** | **Overall** | |
| Cea-Soriano (2012) | ✔ | | ✔ | | ✔ | ✔ | ✔ | ✔ | ✔ | ✔ | ✔ | ✔ | ✔ | ✔ | |
| Gil (2011) | ✔ | | ✔ | | ✔ | ✔ | ✔ | ✔ |  | ✔ | ✔ | ✔ | ✔ | ✔ | |
| Mikkelsen (2021) | ✔ | | ✔ | | ✔ | ✔ | ✔ | ✔ | ✔ | ✔ | ✔ | ✔ | ✔ | ✔ | |
| Weill (2021) | ✔ | | ✔ | | ✔ | ✔ | ✔ | ✔ | ✔ | ✔ | ✔ | ✔ | ✔ | ✔ | |

1. Were the two groups similar and recruited from the same population?

2. Were the exposures measured similarly to assign people to both exposed and unexposed groups?

3. Was the exposure measured in a valid and reliable way?

4. Were confounding factors identified?

5. Were strategies to deal with confounding factors stated?

6. Were the groups/participants free of the outcome at the start of the study (or at the moment of exposure)?

7. Were the outcomes measured in a valid and reliable way?

8. Was the follow up time reported and sufficient to be long enough for outcomes to occur?

9. Was follow up complete, and if not, were the reasons to loss to follow up described and explored?

10. Were strategies to address incomplete follow up utilized?

11. Was appropriate statistical analysis used?
